# Data on RT-qPCR assay of nuclear progesterone receptors (nPR), membrane progesterone receptors (mPR) and progesterone receptor membrane components (PGRMC) from human uterine endometrial tissue and cancer cells of the Uterine Cervix

**DOI:** 10.1016/j.dib.2020.105923

**Published:** 2020-06-25

**Authors:** Natalia Smaglyukova, Elise Thoresen Sletten, Anne Ørbo, Georg Sager

**Affiliations:** a Research group for Experimental and Clinical Pharmacology, Department of Medical Biology, Arctic University of Norway, Tromsø, Norway; b Department of Gynecologic Oncology, Clinic for Surgery, Cancer and Women's Diseases, University Hospital of North Norway, Tromsø, Norway; c Research group for Gynecologic Oncology, Department of Medical Biology, Faculty of Health Sciences, Arctic University of Norway,Tromsø, Norway; d Department of Clinical Medicine, Faculty of Health Sciences, Arctic University of Norway, Tromsø, Norway; e Department of Clinical Pathology, University Hospital of North Norway, Tromsø, Norway; f Clinical Pharmacology, Department of Laboratory Medicine, University Hospital of North Norway, Tromsø, Norway

**Keywords:** RT-qPCR, mRNA, Progesterone, Nuclear receptors, Membrane receptors, Receptor membrane components

## Abstract

A previous investigation showed that the endometrium normalized in women with endometrial hyperplasia after three months treatment with high dose levonorgestrel IUS (intrauterine system) [1] . The effect was maintained even if immunohistochemical analyses of the endometrium showed that nuclear progesterone receptors (nPRs) were completely downregulated. These observations indicated that some type of non-genomic effect existed [2]. We conducted new investigations of endometrial hyperplasia, now with 6 months low dose levonorgestrel IUS treatment. Again, the growth disturbances were reversed with normalization of the endometrium [3,4]. In the context of these studies, RT-qPCR analyses of the endometrium were performed before and after treatment, to determine expression of nuclear progesterone receptors (nPRA+*B* and nPRB), membrane progesterone receptors (mPR, α-, β- and γ-subtypes) and progesterone receptor membrane components (PGRMC1and PGRMC2). The human cervical cell line (C-4 I) [5] with no detectable nPRs [6,7] , was included in the investigation as biological control .The gene expression of nPRs, mPRs and PGRMCs was determined in the logarithmic growth phase. Tissue and cellular mRNA was determined with RT-qPCR and used as a surrogate marker for receptor (protein) expression. The present data are connected to the related article entitled “Expression of nuclear progesterone receptors (nPRs), membrane progesterone receptors (mPRs) and progesterone receptor membrane components (PGRMCs) in the human endometrium after 6 months levonorgestrel low dose intrauterine therapy” [8].

**Specifications Table**

**Table:** 

Subject	*Molecular Biology*
Specific subject area	*mRNA and gene expression of progesterone receptors and receptor membrane components*
Type of data	*Table* *Chart* *Graph*
How data were acquired	*RT-qPCR (Reverse transcription polymerase chain reaction)* *Instruments: Experion™ electrophoresis system (Bio-Rad, Hercules, CA, USA), NanoDrop 2000c spectrometer (Thermo Scientific, Wilmington, DE, USA), CFX96 real-time PCR detection system (Bio-Rad, Hercules, CA, USA), Software: qBase, geNorm*
Data format	*Raw and analyzed*
Parameters for data collection	*Endometrial biopsies from women with endometrial hyperplasia were obtained before end after treatment with levonorgestrel intrauterine device. The human cervical cell line (C-4 I) were expanded and harvested during logarithmic growth. Expression of genes for nuclear progesterone receptors, membrane progesterone receptors and progesterone receptor membrane components were evaluated.*
Description of data collection	*Total RNA were extracted from human endometrium and human C-4I cells to perform RT-qPCR*
Data source location	*UiT - The Arctic University of Norway, Tromsø, Norway*
Data accessibility	*The data are hosted in a public repository (Mendeley Data):https://data.mendeley.com/datasets/4fwxby52h9/1*
Related research article	*E.T.Sletten, N. Smaglyukova. A.Ørbo and G.Sager, Expression of nuclear progesterone receptors (nPR), membrane progesterone receptors (mPR) and progesterone receptor membrane components (PGRMC) in the human endometrium after 6 months levonorgestrel low dose intrauterine therapy, Journal of Steroid Biochemistry and Molecular Biology* *https://doi.org/10.1016/j.jsbmb.2020.105701*

**Value of the Data**

• The regulation of receptors that activate signal pathways of progesterone and gestagens are incompletely understood
• The present data will be useful for scientists in the basic and clinical research of progesterone biochemistry, physiology and pharmacology
• The present data represent a potential tool for scientists to characterize the complex interplay between nuclear progesterone receptors (nPRs), membrane progesterone receptors (mPRs) and progesterone receptor membrane components (PGRMCs) [9,10]
• The data can lead to development of new experiments to extend the knowledge of the regulation of nuclear as well as membrane progesterone receptors under various physiological conditions and under influence of pharmacological substances i*n vivo* and *in vitro*

## 1 Data description

**Fig. 1:**
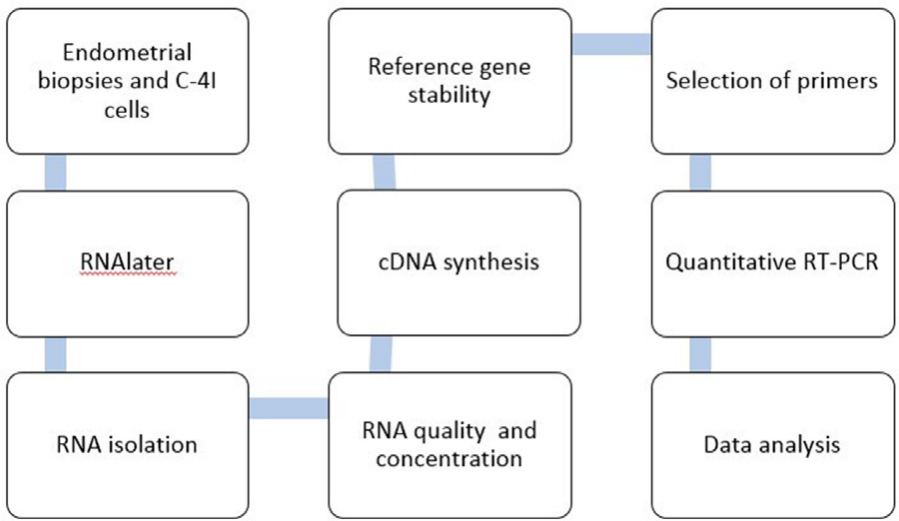
Experimental design for RT-qPCR analysis of nPR (nuclear progesterone receptors), mPr (membrane progesterone receptors) and PGRMC (progesterone receptors membrane components) gene expression (mRNA).

**Fig. 2:**
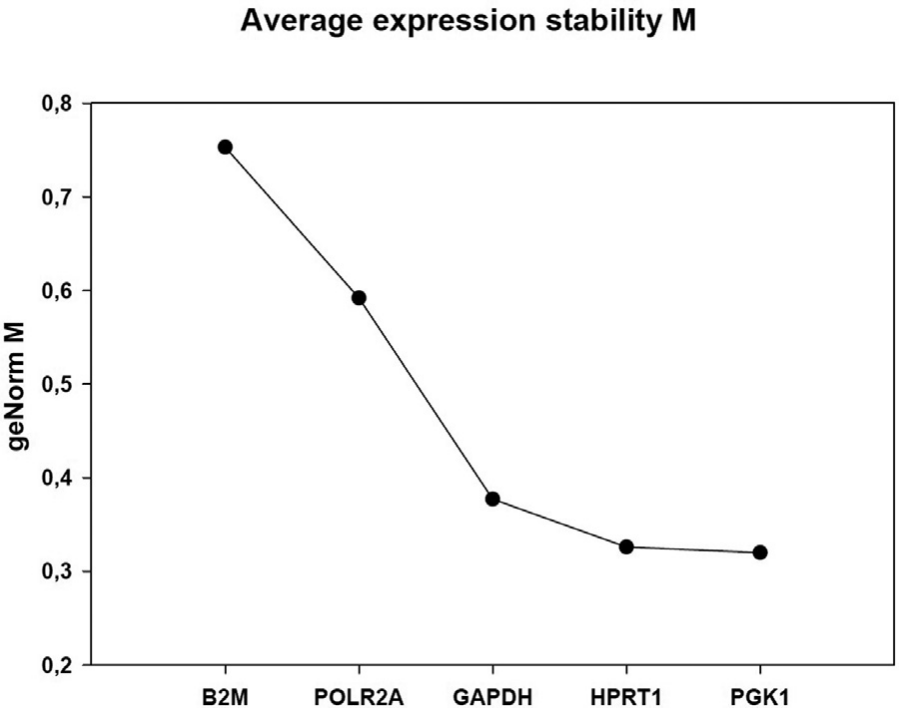
Stability of reference genes tested for endometrial biopsies. B2M (Beta-2-Microglobulin), POLR2A (RNA Polymerase II Subunit A), GAPDH (Glyceraldehyde-3-Phosphate Dehydrogenase), HPRT1 (hypoxanthine phosphoribosyltransferase 1) and PKG1 (protein kinase cGMP-dependent 1).

**Fig. 3:**
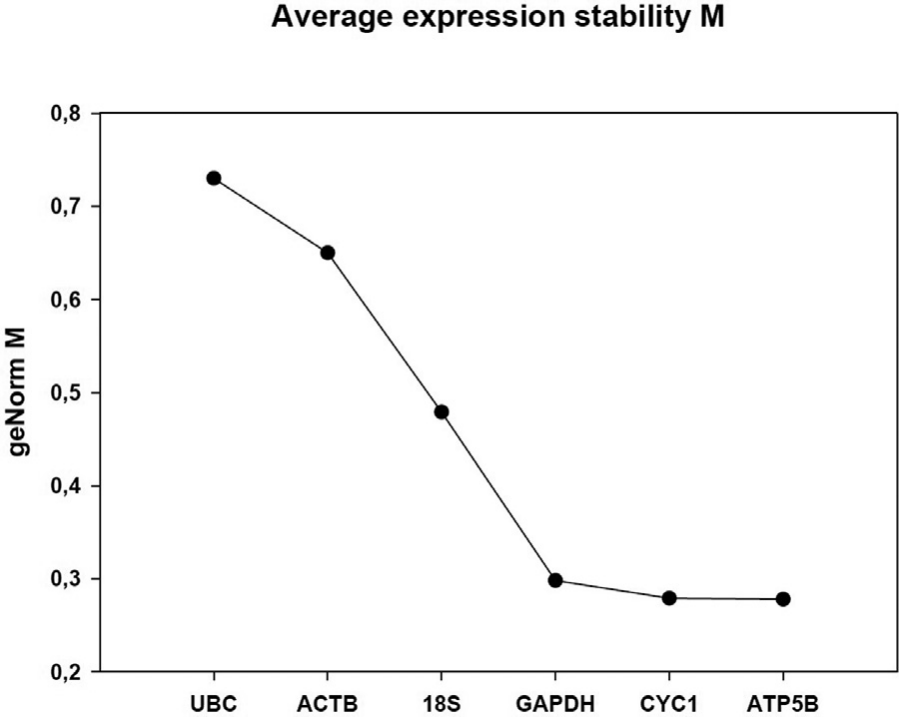
Stability of reference genes tested for C-4I cells. UBC (ubiquitin C), ACTB (Actin Beta), 18S (18S ribosomal RNAs), GAPDH (Glyceraldehyde-3-Phosphate Dehydrogenase), CYC 1 (Cytochrome C1) and ATP5B (ATP Synthase F1 Subunit Beta). .

The present data are a result of the investigation of nPR, mPR and PGRMC gene expression in human endometrium and in a human cancer cell line from the uterine cervix (C-4I) and obtained according to the flowchart in Fig. 1. Table 1 shows the gene symbols, NCBI genBank accession numbers gene names, primer sequences, amplicon size and PCR efficiency. Fig. 2 shows the difference in stability of reference genes tested for the analysis of endometrial biopsies. The order of stability was PKG1 ≥ HPRT1 ≥ GAPDH > POLR2A > B2M. The three most stable (GAPDH, HPRT1 and PGK1) were used for normalization (Table 1). Fig. 3 shows the difference in stability of reference genes tested for the analysis of C-4I cells. The order of stability was ATP5B = CYC 1 = GAPDH > 18S > ACTB > UBC. In the analyses CYC 1 and ATP5B were selected as reference genes for normalization control of C-4I cells (Table 1).

**Table 1:** List of primers for reference genes employed in qPCR-analysis. For the cultured C-4I cells, a commercially available reference gene primer sets CYC 1, ATP5B, GAPDH, 18S, ACTB and UBC were used (Primerdesign Ltd., Southampton, UK).

Gene symbol	NCBI GenBank Accession No.	Gene Name	Primer sequence 5`−3` (F,R)	Amplicon size (bp)	PCR efficiency%
GAPDH	NM_001289745	Glyceraldehyde-3-phosphate dehydrogenase	5′GAGCGAGATCCCTCCAAAAT3′ 5′AAATGAGCCCCAGCCTTCT3’	101	90.8
HPRT1	NM_000194	Hypoxanthine phosphoribosyltransferase 1	5′CTAATTATGGACAGGACTGAAC3′ 5′AGCAAAGAATTTATAGCCCC3′	108	99.1
PGK1	NM_000291	Phosphoglycerate kinase 1	5′CTAAGCAGATTGTGTGGAATG3′ 5′CTCACATGGCTGACTTTATC3′	187	94.9
POLR2A	NM_000937	Polymerase II subunit A	5′GAATACCTTCCACTATGCTG3’ 5′AGAATATCCTTGGCTCTCTC3’	162	–
B2M	NM_004048	Beta-2-microglobulin	5′TCATCCACCAGCAGAGAATGGAA3’ 5′TCTGAATGCTCCAGTTTTTCAA3’	126	–
CYC 1	NM_001916	Cytochrome-c1			95.8
ATP5B	NM_001686	ATP synthase, *H*+ transporting, mitochondrial F1 complex, beta polypeptide			93.7
GAPDH	NM_001289745	Glyceraldehyde-3-phosphate dehydrogenase			–
18S	X03205	18S ribosomal RNA			–
ACTB	NM_001101	Actin beta			–
UBC	NM_021009	Ubiquitin C			–

Table 2 shows the primers used for detection of PRA+PRB, PRB, mPRα, mPRβ, mPRγ, PGRMC1, PGRMC2 by RT-qPCR, in addition to gene symbols, NCBI genBank accession numbers, gene names, primer sequences, amplicon size and PCR efficiency. The corrected normalized relative quantity (CNRQ) was calculated for each gene and scaled to the average value.

**Table 2:** List of primers used for detection of PRA+PRB, PRB, mPRα, mPRβ, mPRγ, PGRMC1, PGRMC2 by RT-qPCR.

Gene symbol	NCBI GenBank Accession No.	Gene Name	Primer sequence 5`−3` (F,R)	Amplicon size (bp)	PCR efficiency%
PRA+PRB	NM_001202474	Homo sapiens progesterone receptor (PGR), transcript variant 1, mRNA	5′AGGTCTACCCGCCCTATCTC3’ 5′TCCCACAGGTAAGGACACCA3’	150	93.5
PRB	NM_000926	Homo sapiens progesterone receptor (PGR), transcript variant 2, mRNA	5′GCACGAGTTTGATGCCAGAGA3’ 5′CTGCGACGGCAATTTAGTGA3’	69	98.4
mPRα	NM_178,422	Progestin and adipoQ receptor family member 7 (PAQR7)	5′CGCTCTTCTGGAAGCCGTACATCTATG3′ 5′CAGACGGTGGGTCCAGACATTCAC3′	121	110.6
mPRβ	NM_133,367	Progestin and adipoQ receptor family member 8 (PAQR8)	5′GTCCATCTGTACGCTCTCCC3′ 5′GCAGGCCATGTGGACAGATA3′	106	95.9
mPRγ	NM_017705	Progestin and adipoQ receptor family member 5 (PAQR5)	5′CAGCTGTTTCACGTGTGTGTGATCCTG3′ 5′GGACAGAAGTATGGCTCCAGCTATCTGAG3′	144	99.8
PGRMC1	NM_006667	Progesterone receptor membrane component 1	5′TGACCTTTCTGACCTCACTGC3’ 5′GCCCACGTGATGATACTTGA3’	85	93.4
PGRMC2	NM_006320	Progesterone receptor membrane component 2	5′TCGAGAATGGGAAATGCAG3′ 5′TTGTGATCCTTGGTATCTTCTTCA3′	111	92.8

## 2 Experimental design, materials, and methods

### 2.1 Experimental design

Fig. 1 shows a flowchart for the experimental design of RT-qPCR analysis of nPRs (nuclear progesterone receptors), mPRs (membrane progesterone receptors) and PGRMCs (progesterone receptor membrane components) gene expression (mRNA).

### 2.2 Materials

Endometrial biopsies with analyzable tissue samples were obtained from 42 women. A group of women (*n* = 61) was recruited to a prospective, multicenter pilot investigation to assess the efficacy of LNG-IUS 13.5 mg (Jaydess™, Bayer Pharmaceuticals, Berlin, Germany) for treatment of endometrial hyperplasia. Only those women (*n* = 49) with a completed treatment period of six months were included in the present work. In seven of the 49 women, endometrial biopsy material was insufficient for qPCR analysis. Endometrial biopsies were obtained by an endometrial suction cuvette (Pipelle™, Laboratoire CCD, Paris, France) or dilatation and curettage or hysteroscopic transcervical resection. All endometrial tissue specimens (baseline and post therapy biopsies) were conserved in RNAlater stabilization solution (Ambion RNAlater™, ThermoFisher Scientific, Whatham, MA, USA, code AM7021) and immediately frozen and kept at-18 °C until further analysis. Details of the clinical investigations are reported earlier [3,4] .

The human cell line C-4I (ATCC, CRL­1594™) was derived from a squamous carcinoma of the uterine cervix [5] and obtained from the American Type Culture Collection (Rockville, MD, USA). The cells were cultured in RPMI-1640 (Sigma-Aldrich, St. Louis, MO, USA, code R8758) with 10% (v/v) fetal bovine serum (Sigma-Aldrich, St. Louis, MO, USA, code F7524 ), 1% Penicillin-Streptomycin with 10,000 units penicillin and 10 mg streptomycin per mL in 0.9% NaCl (Sigma-Aldrich, St. Louis, MO, USA, code P0781) at 37 °C in a humidified 5% CO_2_ atmosphere in our laboratory. The cells were seeded in six well plates at a density of 4 × 10^4^ cells/mL in five parallels. The medium was changed every second day and the cells were harvested in the logarithmic phase of growth (five days after seeding).

To collect the cultures for RNA extraction, media was removed by aspiration, and the cells were brought into a suspension using 200 μL 0.25% (w/v) trypsin with 0.2% (w/v) Na_4_EDTA, (Sigma-Aldrich, St. Louis, MO, USA, code T4049), and the trypsin activity was terminated by addition of 800 μL of incubation media. The cell counts were obtained (Contess Automated Cell Counter™, Invitrogen, ThermoFisher Scientific, Carlsbad, CA, USA) and the cells were conserved in RNA stabilization solution (Ambion RNAlater™^,^ ThermoFisher Scientific, Whatham, MA, USA, code AM7021), stored at 4 °C overnight and then transferred to −20 °C until further analysis.

### 2.3 Methods

The first step was RNA isolation and cDNA synthesis. Total RNA extraction from endometrial tissue samples and C-4I cells was performed using a commercial RNA extraction kit (Bio-Rad Aurum Total RNA Mini Kit, BIO-RAD, Hercules, CA, USA, code 732–6820). RNA was isolated according to manufacturer's instruction manual. Tissue samples (10–30 mg) were transferred to MagNA Lyser Green Beads tubes (Roche Molecular Biochemicals, Mannheim, Germany). Lysis buffer (700 µL) from RNA isolation kit, containing 1% (v/v) β-mercaptoethanol (BioRad, Hercules, CA, USA, code 1,610,710), was added to the tubes. The samples were homogenized in a Precellys™ 24 high-throughput homogenizer (Bertin Technologies, Rockville, MD, USA) at 6000 × *g* for 30 s and cooled on ice for 2 min. Afterwards, the samples were left at room temperature for 5 min. RNA was eluted and stored at −70 °C.

RNA quality was evaluated with the Experion™ electrophoresis system (Bio-Rad, Hercules, CA, USA) using the Experion™ RNA StdSens Analysis Kit (Bio-Rad Laboratories, Hercules, CA USA, code 7,007,104). RNA samples isolated from patient biopsies and from cultured cells were examined for concentration, purity and integrity. The concentrations and the purity of RNA were determined using NanoDrop 2000c spectrometer (Thermo Scientific, Wilmington, DE, USA). The endometrial RNA samples showed A260/280 ratio of 2.1 ± 0.023 (mean ± SD) and a RNA integrity of 8.5 ± 0.9 (mean ± SD). The A260/280 ratio of the C-4I cell RNA samples was 2.1 ± 0.012 (mean ± SD) and with a RNA integrity of 9.9 ± 0.1 (mean ± SD).

The concentration of RNA was measured using NanoDrop 2000c spectrophotometer (Thermo Scientific, Wilmington, DE, USA). Reverse transcription was performed using iScript™ cDNA Synthesis Kit (Bio-Rad, Hercules, CA, USA, code 170–8891). A total of 500 ng RNA was reverse transcribed in a final volume of 20 µL. After the synthesis, cDNA was diluted 10 x with nuclease free water (Promega, Madison, WI, USA, code P1195). The cDNA was stored at −20 °C for further use.

The next step was analysis of reference gene stability, selection of primers and reaction efficiency. The most stable reference genes were chosen for normalization calculations in qBase program. Predesigned reference genes were obtained from Sigma-Aldrich (Haverhill, UK) and Primerdesign Ltd (Southampton, UK). The geNorm program (https://genorm.cmgg.be) was used to find the most stable gene transcript of reference genes for normalization controls. In the qPCR analyses, several reference genes belonging to different functional classes were evaluated by the geNorm software. For the endometrial biopsies five reference genes (B2M, POLR2A, GAPDH, HPRT1 and PGK1) were examined. Six genes (UBC, ACTB, 18S, GAPDH, CYC1 and ATP5B) were evaluated as reference genes for C-4I cells. Pairwise variation (Vn/*n* + 1) geNorm V analysis was carried out to determine the number of reference genes required for normalization. In the case of C-4I cells, the optimal number was two reference genes, Cyc1 and ATP5B.

PRA+PRB, PRB and mPRβ primers were designed using Primer-blast on the NCBI website (http://blast.ncbi.nlm.nih.gov/). The design of mPRα and mPRγ primers was performed as described by Dressing and Thomas [11] and those for PGRMC1 and PGRMC2 as described by Causey et al. [12].The sequences of primers are listed in Table 2.

A standard curve from a twofold serial dilution of pooled cDNA was made and gave a high RT-qPCR reaction efficiency of the primer pairs, shown in Table 1 and Table 2, respectively. A gene-specific amplification was confirmed by a single peak in the melting curve analysis.

Real-time qPCR was carried out using the CFX96 real-time PCR detection system (Bio-Rad, Hercules, CA, USA) and SsoAdvance Universal SYBR Green Supermix (BIO-RAD, Hercules, CA, USA, code 172–5271) and human SYBRgreen reference gene detection kit. 900 reaction (Primerdesign Ltd., Southampton, UK, code HK-SY-hu-900). The assay was run with 2.5 µL of 10 × diluted cDNA in a 10 µL total reaction supermix. The thermal profile for SYBR real-time qPCR included an initial heat-denaturing step at 95 °C for 30 s, 40 cycles at 95 °C for 5 s and 60 °C for 30 s. Following amplification, a melt-curve analyses of the PCR products were performed from 65 °C to 95 °C to determine the specificity of amplification. Each sample was run in duplicate. A non-template control and an inter-run calibrator were added to each run. Data acquisition and subsequent data analyses were performed using the CFX Manager software (Bio-Rad, Hercules, CA, USA). Gene expression was analyzed with qBase software as described by Hellemans et al. [13] .The program employs a modified delta-Ct method with the possibility to adjust for PCR efficiency and to use multiple reference genes for normalization. The corrected normalized relative quantity (CNRQ) was calculated for each gene and scaled to the average value.

## Declaration of Competing Interest

None.
